# Supplementary material for: Retinal Responses to Visual Stimuli in Interphotoreceptor Retinoid Binding-Protein Knock-Out Mice
Source: Int J Mol Sci. 2023 Jun 26;24(13):10655. doi: 10.3390/ijms241310655 (PMC10341985; doi:10.3390/ijms241310655)
Supplement: Supplementary file 1 [file ijms-24-10655-s001.zip › ijms-2362495-supplementary.pdf]

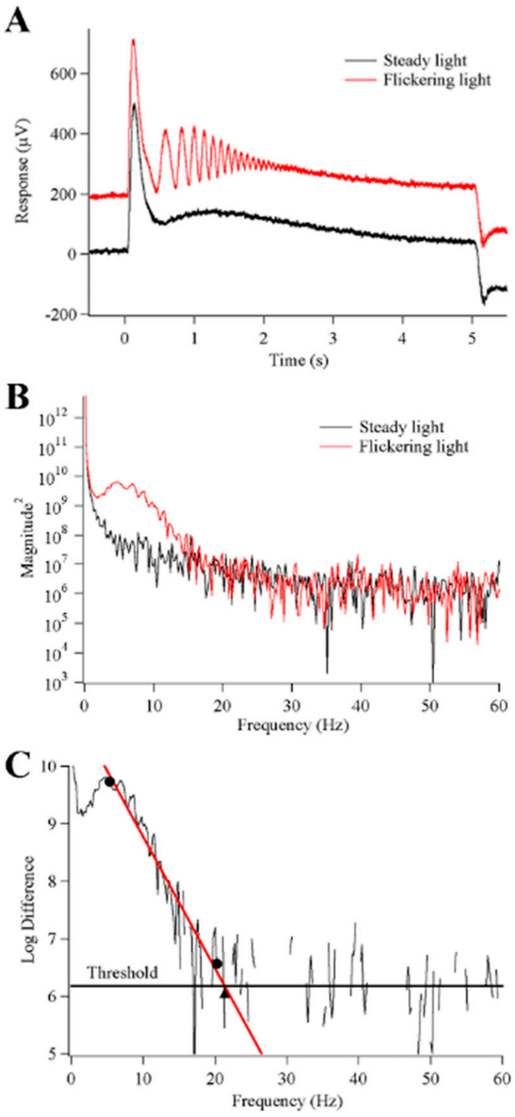

Figure S1. Photopic flicker sweep ERG responses and calculations of CFF. A. Steps of flickering light were interleaved with steady steps of light of the same mean intensity and duraton. B. The FFT of the light responses across the 5 s stimulus shows the entire frequency response of the ERG. C. The difference spectra allow a linear regression fitting on a log-difference vs frequency graph. Threshold was previous established as 6.18 Log and allows an estimate of the CFF. Stimulus swept from 0.1-30Hz for scotopic and 0.1-55 Hz for photopic tests; recorded DC 300Hz; mean power of 2.40 mW/s; 94% contrast). (WT n=12, IRBP KO n= 13). Analyses detailed in methods.
